# Differential Response of Mycorrhizal Plants to *Tomato bushy stunt virus* and *Tomato mosaic virus* Infection

**DOI:** 10.3390/microorganisms8122038

**Published:** 2020-12-19

**Authors:** Neda Khoshkhatti, Omid Eini, Davoud Koolivand, Antreas Pogiatzis, John N. Klironomos, Sepideh Pakpour

**Affiliations:** 1 Department of Plant Protection, Faculty of Agriculture, University of Zanjan, Zanjan 45195-313, Iran; nkhoshkhati@gmail.com (N.K.); omid.eini@znu.ac.ir (O.E.); Koolivand@znu.ac.ir (D.K.); 2 Department of Biology, University of British Columbia, Kelowna, BC V1V1V7, Canada; antreas.pogiatzis@ubc.ca; 3 School of Engineering, University of British Columbia, Kelowna, BC V1V1V7, Canada

**Keywords:** gene expression, mycorrhization, *Rhizoglomus irregulare*, *Tomato mosaic virus*, *Tomato bushy stunt virus*

## Abstract

*Tomato bushy stunt virus* (TBSV) and *Tomato mosaic virus* (ToMV) are important economic pathogens in tomato fields. *Rhizoglomus irregulare* is a species of arbuscular mycorrhizal (AM) fungus that provides nutrients to host plants. To understand the effect of *R. irregulare* on the infection by TBSV/ToMV in tomato plants, in a completely randomized design, five treatments, including uninfected control plants without AM fungi (C), uninfected control plants with AM fungi (M) TBSV/ToMV-infected plants without AM fungi (V), TBSV/ToMV-infected plants before mycorrhiza (VM) inoculation, and inoculated plants with mycorrhiza before TBSV/ToMV infection (MV), were studied. Factors including viral RNA accumulation and expression of Pathogenesis Related proteins (PR) coding genes including *PR1*, *PR2*, and *PR3* in the young leaves were measured. For TBSV, a lower level of virus accumulation and a higher expression of PR genes in MV plants were observed compared to V and VM plants. In contrast, for ToMV, a higher level of virus accumulation and a lower expression of PR genes in MV plants were observed as compared to V and VM plants. These results indicated that mycorrhizal symbiosis reduces or increases the viral accumulation possibly via the regulation of PR genes in tomato plants.

## 1. Introduction

A mutualistic association exists between arbuscular mycorrhizal (AM) fungi and 85% of land plants with substantial advantages to plant fitness and growth [1,2]. Increased productivity and biomass of AM fungi colonized plants is not only the result of enhanced mineral nutrition [3,4], but also the result of improved capability to deal with both abiotic and biotic stresses [2,4,5]. For example, the reduction of symptoms in mycorrhizal plants were observed compared to control plants when they were infected by fungi such as *Alternaria solani* [5] and *Botrytis cinerea* [6,7], or bacteria such as *Xanthomonas campestris* [8] and phytoplasma [9,10]. However, scientific research has revealed that colonization of plants by AM fungi may have positive or negative effect on viral infections [11]. *Solanum lycopersicum* (tomato) has developed a range of sophisticated defense mechanisms, induced by either non-pathogenic micro-organisms or environmental factors prior to disease development [7] when reacted to viral pathogens. For example, Maffei et al. [11] found that AM-colonized tomato plants had reduced viral titer when infected by *Tomato yellow leaf curl Sardinia virus* (TYLCSV). However, AM colonization could not confront root biomass reduction created by the virus. In contrast, Jabaji-Hare and Stobbs [12] reported viral titer increase in TMV infected plants colonized by *Glomus* sp. Similar results were obtained in the leaves and roots of tomato plants infected with *Potato virus X* (PVX) colonized by *Funneliformis macrocarpa* [13]. Likewise, Miozzi et al. [14] examined the interactions between *Tomato spotted wilt virus* (TSWV) and *Funneliformis mosseae* (syn. *Glomus mosseae*) in tomato plants and found that the expression level of defense-related genes was reduced by mycorrhization and consequently a higher virus titer was observed in mycorrhizal plants.

Viral infections cause loss in tomato yield production worldwide [15,16] and *Tomato bushy stunt virus* (TBSV) and *Tomato mosaic virus* (ToMV) are two of the causal agents of such diseases [17]. However, the effect of AM fungi on the pathogenicity of these viruses and underlying mechanisms affecting tomato plant infection is unknown.

Susceptibility or resistance of the different mycorrhizal plants against the virus might be associated with the pathogenesis related (PR) proteins. PR proteins are intra and extracellularly localized proteins produced in plants followed by pathogen attack. They are induced as part of systemic acquired resistance (SAR) [18]. Song et al. [19] found a robust induction of PR protein genes (*PR1*, *PR2* and *PR3*) in mycorrhizal tomato plants in comparison to non-mycorrhizal plants in the presence of fungal infection. However, when a virus infected the mycorrhizal tomato plants, namely the *Tomato spotted wilt virus* (TSWV), lower levels of PR proteins were detected [20]. Similarly, reduction in the number of PR proteins (*PR1* and *PR3*) coding genes in mycorrhizal tobacco plants infected with *tobacco mosaic virus* was observed [21].

Our knowledge on whether root colonization by AM fungi has a protective or destructive effect during TBSV or ToMV viral infection is still limited. Our study showed the impact of the AM fungi symbiosis on the severity of viral infection in tomato plants and elucidated the relationship between viral resistance and PR protein expression levels in response to AM fungi colonization.

## 2. Materials and Methods

### 2.1. Experimental Design and Biological Materials

Fungal spores of *R. irregularis* were provided by AGTIV, Premier TechAgriculture Canada and viruses were provided by Agri-Food and Agriculture Canada institute. Tomato seeds were placed in germination trays containing vermiculite and sand. Four weeks post planting, the seedlings were transferred to 1 L pots containing vermiculate soil and sand at a ratio of (1:1, *w*/*w*). Using a fully randomized experimental design, five treatment groups were included: (i) uninfected control plants without AM fungi (C), (ii) uninfected control plants with AM fungi (M), (iii) TBSV/ToMV-infected plants without AM fungi (V), (iv) TBSV/ToMV-infected plants before mycorrhiza (VM), and (v) inoculation and inoculated plants with mycorrhiza before TBSV/ToMV infection (MV). For each treatment, there were three replicates.

About 150 mL from spray suspension of *R. irregularis* [1 g (12,000 viable spores per gram of powder) in 1 L] near the roots was used to inoculate the MV and M treatments. Plants were grown in a greenhouse and were exposed to a cycle of 14:10 h light:dark at a temperature of 23 ± 3 °C. The plants were sprayed once a week with a nutrient solution. MV plants was inoculated with viruses, 20 days after inoculation with AM fungi. For the VM group, ten days after virus inoculation, plants were inoculated with *R. irregularis*. Plants were then investigated for symptom development at three stages of 20, 24, and 30 days post inoculation (dpi) with the virus (Table 1).

### 2.2. Viral Affirmation and Quantification

Total RNA was extracted from young leaves of C, M, MV, V, and VM plants at 20, 24 and 30 dpi by using RNeasy Mini Kit (Qiagen, Hilden, Germany). It is known that virus accumulation varies in different tissues and leaves with different ages [22]. Since the effect of age was not the focus of our study, and to avoid possible variation among plant tissues for virus accumulation and gene expression, all tests were conducted on only a single young leaf (second leaf from top) for each plant. In addition, to prevent potential effect form wounding during sampling on plant gene expression, tissue samples were freezed in liquid nitrogen immediately after collection [23,24]. The samples were then grinded to obtain a homogenized powder, and then RNA was extracted from an individual new leaf (~100 mg) for each treatment.

*DNase* I kit (Ambion, Austin, TX, USA) was used to remove DNA contaminations following the manufacturer’s instructions. The purified RNA was used for Random hexamer-primed first-strand cDNA synthesis by iScript cDNA Synthesis Kit (BioRad, Hercules, CA, USA). Specific primer pairs [25], corresponded to TBSV-Gral-R1 (5′-TTTGGTAGGTTGTGGAGTGC-3′) and TBSV-Gral-F1 (5′-AAGGGTAAGGATGGTGAGGA-3′) were used to quantify the TBSV virus in test samples. For ToMV [26], the ToMVR (5′-GACCCCAGTGTGGCTTCGT-3′) and ToMVF (5′-TTGCCGTGGTGGTGTGAGT-3′) primers were used. For each test, 2 μL of template cDNA was utilized in a reaction, including 10μL of SsoFastGreen supermix buffer and 0.6 μL of primers: (TBSV-GRAL for TBSV) and (ToMVF and ToMVR for ToMV). In order to examine the reaction specificity, at the end of each run a melting curve was recorded. The viral accumulation level was normalized to that of a reference gene [19], *UBI3* (Accession No. X58253), utilizing LeUBI3 primers (Table 2). The $2^{-∆∆C_{t}}\;$ method as described by Livak and Schmittgen [27] was used to calculate the relative accumulation of the virus for each sample. Three biological replicates were performed for each treatment. Statistical analysis of the mean of biological replicates at three time points was performed by repeated measures ANOVA and Bonferroni post-hoc test utilizing SPSS software. We also used ANOVA-Tamhan post-hoc test for comparing viral expression between five treatments.

### 2.3. Disease Severity Evaluation

Symptoms in the inoculated plants were observed, beginning in the second week and assessed at 20, 24, 30 dpi in V, MV and VM plants. In the infected plants, disease symptoms were scored by utilizing the following scale as Friedmann et al. [28] suggested, with some modifications; for infected plants with TBSV: zero for having no symptoms; one for leaf mild mosaic; two for leaf mosaic and cupped leaf; three for yellowing, cupped leaf; four for yellowing, cupped leaf and stunted plants. For infected plants with ToMV: zero for having no symptoms; one for leaf mild mosaic; two for leaf mosaic and leaf deformation; three for yellowing, leaf deformation; and four for yellowing, leaf deformation and stunted plants. The index of plant disease severity (PDS) was calculated, as previously described [29,30]. Briefly, PDS = Sum of numerical rating/ (total number of observed × maximum disease grade) × 100. Also, we used an analysis of variance for the calculated PDS to differentiate (ANOVA-Bonferroni post hoc-test, *p* **<** 0.05) the response of each treatment to viruses statistically.

### 2.4. Differential Gene Expressions

The expression patterns of the three pathogen-related genes (*PR1*, *PR2*, and *PR3*) were analyzed using RT-qPCR from tomato young leaves at 20, 24 and 30 dpi (days post infected with virus). RT-qPCR tests were performed for these genes, utilizing the prepared cDNA and their specific primers (Table 2). Each reaction included 2 μL of cDNA template, 1 μL of each primer, and 10 μL SsoFastGreen super mix (Bio-Rad). The PCR cycling program contained: 95 ºCfor 10 min, followed by 40 cycles at 95 °C for 20 s, 56 °C for 30 s, 72 °C for 30 ending with a melting curve from 60 to 95 °C. The efficiency of PCR was measured by drawing a standard curve for each gene and preparing serial dilutions of pooled cDNAs. The expression level of these genes for each sample was normalized to that of the reference gene, *UBI* (ubiquitin). Calculating and analyzing the relative amount of gene expression for each sample was performed, as described above.

### 2.5. Mycorrhiza and Biomass Assessment

To avoid possible stress applying to plant roots (MV, VM and M), we only assessed mycorrhization at the end of experiments. For measuring AM colonization, M, VM and MV plants were carefully pulled up (with their whole roots) from each treatment and rinsed with water to clean sticky soil particles. Modified Vierheilig et al. [31] Sheaffer ink protocol was used to stain roots. Briefly, the roots were rinsed for 5 min in 10% KOH at 90 °C and then washed three times with RO water. Cleared roots were stained in 5% black Sheaffer Ink and vinegar solution at 90 °C for 3.5 min. RO water with few drops of vinegar was used to de-stain the roots (30 min). Root segments of the whole root system for 8 plants were placed on slides and observations were performed for the presence of vesicles, mycelium or arbuscules according to the Trouvelot quantification method [32]. We did data analysis for calculation of colonization using one-way ANOVA and Bonferroni post-hoc test (*p* < 0.001).

For the evaluation of biomass, the aboveground parts of plants from all treatments were harvested at 80 days after planting the seeds. We determined fresh and dry weigh and conducted statistical analysis on the data (one-way ANOVA-Bonferroni post hoc-test, *p* < 0.05).

## 3. Results

### 3.1. Phenotypic Responses of Mycorrhizal Plants to VIRAL Infection

**Figure 1:**
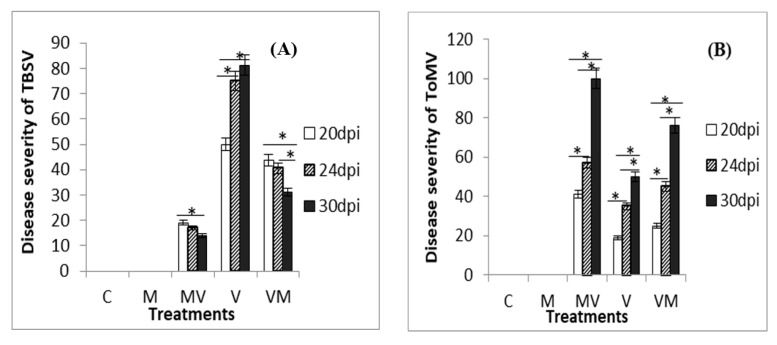
Disease severity of tomato plants infected by TBSV (**A**) and ToMV (**B**) at 20, 24 and 30 dpi (days post inoculation) of uninfected control plants without AM fungi (C), uninfected control plants with AM fungi (M) TBSV/ToMV-infected plants without AM fungi (V), TBSV/ToMV-infected plants before mycorrhiza (VM) inoculation and inoculated plants with mycorrhiza before TBSV/ToMV infection (MV). DS = Disease Severity for three replicates calculated. Significant difference between the disease severity of treatments at three times is shown by (*) [using repeated measures ANOVA and Bonferroni post-hoc test (*p* < 0.05)]. Error bars represent SD.

Various symptoms appeared in both mycorrhizal and non-mycorrhizal plants inoculated by viruses. Infected plants with TBSV indicated leaf mosaic, yellowing, cupped leaf, necrosis and stunting. A significantly higher PDS was observed for V plants as compared to the MV and VM plants at 20, 24 and 30 dpi [ANOVA-Tamhan post-hoc test (*p < 0.05*)]. Also, our results showed a significant decrease of disease severity in MV and VM plants over time [repeated measures ANOVA and Bonferroni post-hoc test (*p* < 0.05)] (Figure 1A).

For ToMV, infected plants showed leaf mosaic and leaf deformation, yellowing and stunting. A significantly higher PDS was seen for MV plants as compared to the V and VM plants at all three times (ANOVA-Tamhan post-hoc test (*p* < 0.05)). A significant increase of disease severity in all treatments was seen over time [Repeated measures ANOVA and Bonferroni post-hoc test (*p* < 0.05)] (Figure 1B). No disease symptoms were observed in the control plants.

### 3.2. Effect of Mycorrhiza on the Viral Accumulation

**Figure 2:**
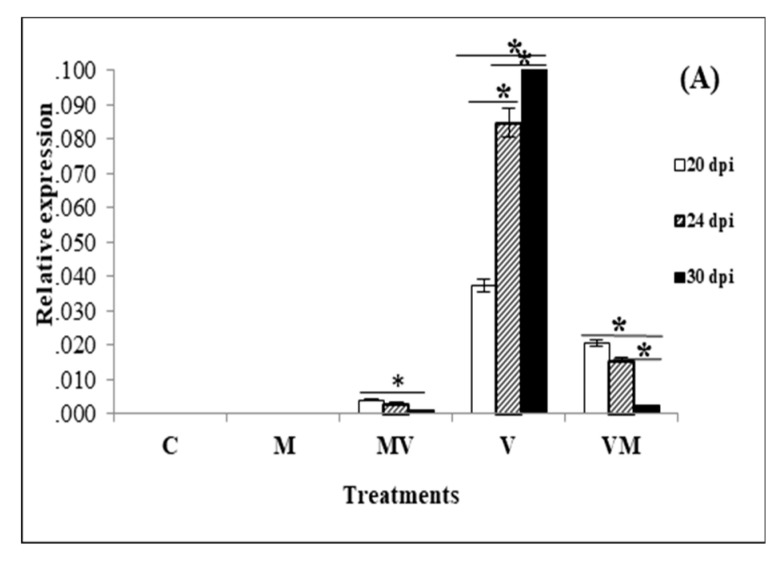

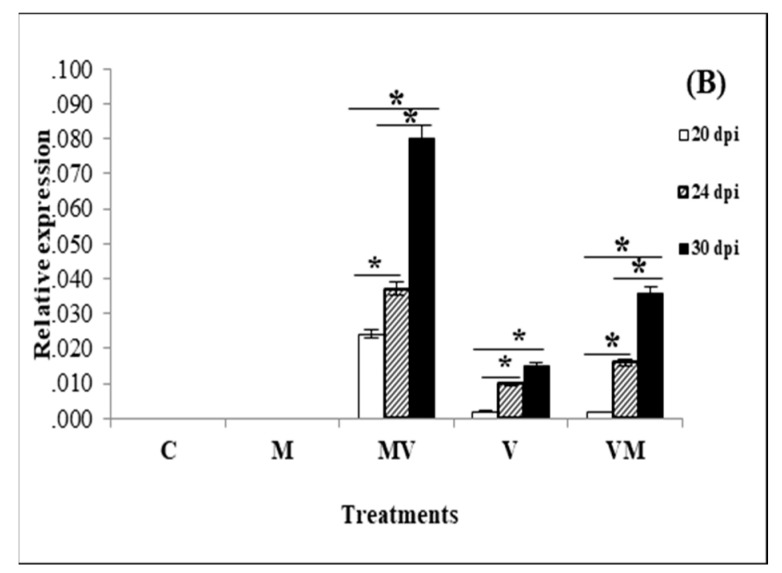
The result of RT-qPCR shows an accumulation level of viral infection by TBSV (**A**) and ToMV(**B**) in uninfected control plants without AM fungi (C), uninfected control plants with AM fungi (M), TBSV/ToMV -infected plants without AM fungi (V), TBSV/ToMV -infected plants before mycorrhiza (VM) inoculation and inoculated plants with mycorrhiza before TBSV/ToMV infection (MV) at three stages of infection (20, 24 and 30 dpi). For each treatment, three replicates were tested by qPCR. A significant difference between the expressions of viral gene for each treatment in three different times is shown by (*) [using repeated measures ANOVA and Bonferroni post-hoc test (*p* < 0.001)]. Error bars represent SD.

Viral accumulation was examined by RT-qPCR in the newly appeared leaf tissues of plants at 20, 24 and 30 dpi. Since the time of simultaneous onset of viral symptoms and mycorrhization was on the twentieth day after inoculation of the plants with viruses, we tested virus accumulation and gene expression assay from 20 dpi to provide a reasonable time for interaction among mycorrhiza, plant and virus. When we compared the TBSV accumulation in the V, MV and VM plants by RT-qPCR, our results showed that V plants produced a significantly higher level of viral accumulation compared to VM and MV plants at 20, 24 and 30 dpi (*p* < 0.001) and the MV plants had the lowest viral accumulation at all-time points. Interestingly, our results demonstrated that TBSV infection was increased over time when plants were not colonized by AM fungi (V plants), while infection had a decreasing trend in AM colonized groups, more significantly in those that were colonized with AM fungi after viral infection (VM group) (Figure 2A). Comparing the ToMV accumulation in the V, MV and VM plants by qPCR demonstrated that MV plants produced a significantly higher level of ToMV accumulation compared to V and VM plants at 20, 24 and 30 dpi [ANOVA-Tamhan post-hoc test (*p* < 0.001)]. Our results also showed that ToMV infection increased significantly over time in the AM colonized groups and in the plants that were not colonized by AM fungi (V plants) [repeated measures ANOVA and Bonferroni post-hoc test (*p* < 0.001)] (Figure 2B).

### 3.3. Regulation of Genes in Mycorrhizal Plants Infected by Viruses

**Figure 3:**
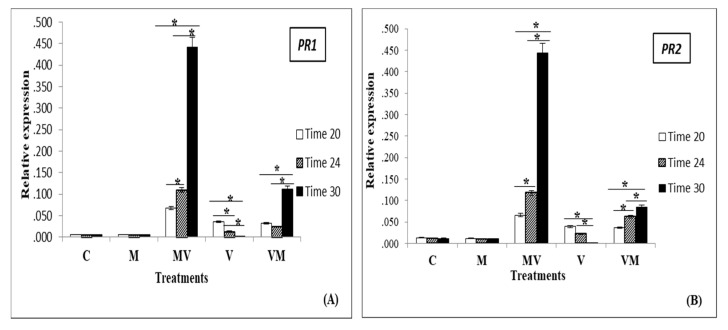

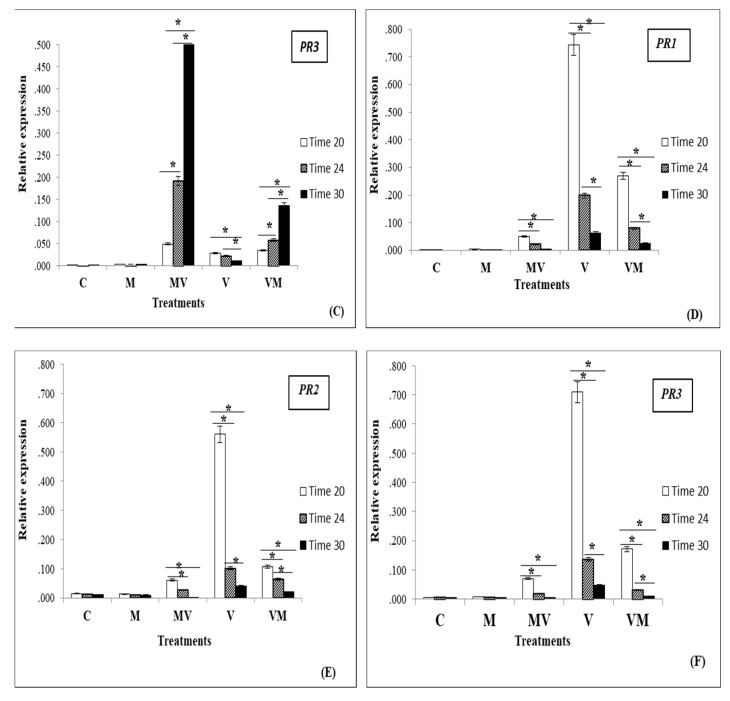
Expression analysis of *PR1*, *PR2*, PR3 genes by RT- qPCR for the plants infected by TBSV (**A**–**C**) and for the plants infected by ToMV (**D**–**F**). Uninfected control plants without AM fungi (C), uninfected control plants with AM fungi (M), TBSV/ToMV -infected plants without AM fungi (V), TBSV/ToMV-infected plants before mycorrhiza (VM) inoculation and inoculated plants with mycorrhiza before TBSV/ToMV infection (MV) at three stages of infection (20, 24 and 30 dpi). For each treatment, three replicates were tested. A significant difference between the expression of PR genes for each treatment in three different times is shown by (*) [using repeated measures ANOVA and Bonferroni post-hoc test (*p* < 0.001)]. Error bars represent the SD.

TBSV and ToMV infection in tomato plants induced the expression of PR genes in the tomato young leaves. For TBSV, the induction was much less and slower in V plants compared to that in the AM colonized groups. Figure 3 showed a significant decrease of expression in *PR1*, *PR2*, and *PR3* in V plants over time. However, this trend was positive in the MV and VM plants (Figure 3A–C). For ToMV, our results showed a significant decrease of expression in PR genes in MV, V and VM plants over time but the induction was significantly much more in V plants compared to that in the AM colonized groups (Figure 3D–F). The results indicated a very low expression of these genes in C and M plants compared to infected groups.

**Figure 4:**
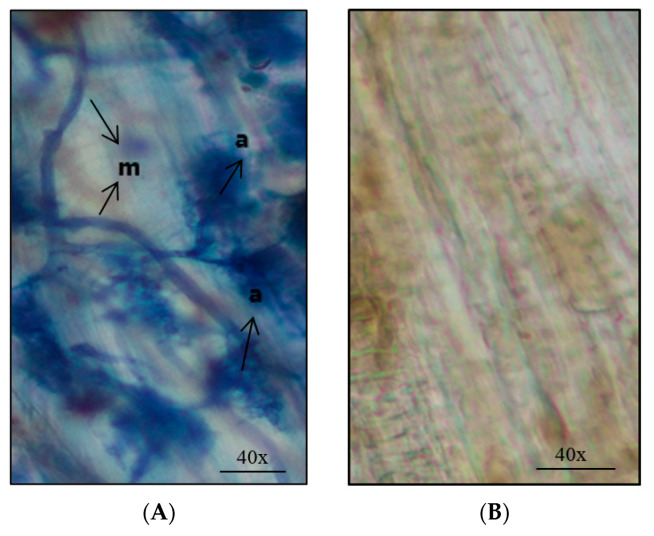
Microscopic image of (**A**) the presence of mycelium (m) and arbuscule (a) of AM fungi in the tomato root and (**B**) its absence in control samples.

**Figure 5:**
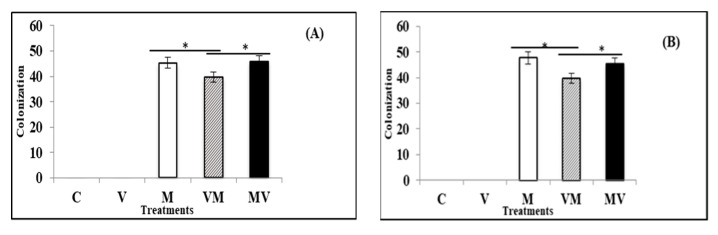
Average percentage of colonization in the plants infected by TBSV (**A**) and ToMV (**B**). Uninfected control plants without AM fungi (C), uninfected control plants with AM fungi (M), TBSV/ToMV -infected plants without AM fungi (V), TBSV/ToMV -infected plants before mycorrhiza (VM) inoculation and inoculated plants with mycorrhiza before TBSV/ToMV infection (MV). Significant difference between colonization of treatments is shown by (*) [using oneway ANOVA and Bonferroni post-hoc test (*p* < 0.001)]. For each treatment, three replicates were tested. Error bars represent SD.

At the end of the experiment, colonization of AM fungi (Figure 4) was evaluated in M, MV and VM plants. For the plants infected by TBSV and ToMV, the percentages of root colonization by *R. irregulare* were about 40%, 46% and 48% in VM, MV and M groups (Figure 5A,B). ANOVA test results showed that there was no significant difference between mean colonization percentage of uninfected control plants with AM fungi (M) and inoculated plants with mycorrhiza before TBSV/ToMV infection (MV). It also showed that the mean of colonization percentage of M and MV treatments were significantly higher than the mean of colonization in the VM treatment (*p* < 0.001).

For TBSV, VM and MV plants produced significantly higher Fresh weight (FW) and Dry weight (DW) compared to the V plants (Table 3). In contrast to ToMV, V plants produced significantly higher FW and DW compared to the MV and VM plants (Table 3). ANOVA test results showed that there was no significant difference between the level of FW and DW in uninfected control plants with AM fungi (M) and uninfected control plants without AM fungi (C) (Table 3).

## 4. Discussion

AMs have been reported to induce host plant resistance against several plant diseases such as below-ground and shoot pathogens, soil-borne fungal pathogens, nematodes and some viruses [33,34,35,36].

Our results suggest that inoculation of tomato plants with *R. irregulare* fungi significantly decreases the accumulation of TBSV RNA in young plant leaves. This was also in accordance with the overall level of biomass in MV and VM treated plants. We observed a statistically higher level of biomass in these plants compared to V treated plants. In a previous study by Maffei et al. [11], a decreased symptom severity and a lower level of accumulation of tomato yellow leaf curl Sardinia virus was reported in AM colonized tomato plants, which may provide a possible explanation of our observed experimental results. On the contrary, while investigating the effect of ToMV infection, our results revealed that ToMV established higher levels of virus accumulation in the young leaves. Plant disease severity (PDS) was also found to be significantly higher for MV treated plants in comparison to the V and VM treated plants at all dpi time points. This is in agreement with previous studies that reported and positive effect of *Piriformospora indica*, on the accumulation of Tomato spotted wilt virus [14] and Pepino mosaic virus [37]. In addition, another study highlighted that a higher level of virus accumulation was found in mycorrhizal petunia (*Petunia atkinsiana)*, tobacco (*Nicotiana tabacum)* and tomato plants affected by Alfalfa Tobacco mosaic virus, mosaic virus, and Potato virus x, respectively [13].

Mycorrhizal colonization is restricted to plant roots. However, its physiological influence may extend beyond to the aboveground non-colonized section of the plant. This systemic effect can be deciphered through gene expression levels in the mycorrhizal plants’ aboveground tissues. For instance, a large number of genes that are associated with defense or stress response were up-regulated in the mycorrhizal Medicago truncatula shoots [8]. Nevertheless, in mycorrhizal tomato plants, PR encoding genes were reported to be down-regulated [21]. The PR proteins were discovered in various plant species [38]. Examples of PR proteins include: PR2 (b-1, 3-glucanase), PR3 (chitinase type 1, 2, 4, 5, 6, 7), PR5 (osmotins), PR1 (unknown), PR10 and PR8 (chitinase type 3) [39,40]. These PR proteins can be produced under a controlled PR genes expression mechanism against related pathogen attack. It has been previously reported that the protein isoforms PR1, PR2, and PR5 are specifically activated during infection with the pepper mild mottle virus (PMMoV) [41]. In another study, viral infection with the Beet severe curly top virus (BSCTV) amplified the expression of several genes that plays a vital role in the salicylic acid pathways, including the PR1 gene [42]. Friorilli et al. [20], in a similar experiment, observed that PR protein expression decreased in the plants colonized with *Glomus mosseae.*

In this study, AM inoculated tomato plants infected with TBSV expressed higher levels of PR gene expression in the young leaves than other treated groups. However, for the inoculated plants infected with ToMV, our findings showed lower levels of PR genes expression in the young leaves of MV plants than V and VM plants. Similarly, in a study by Ebrahimi et al. [43], a reduction in gene expression of the PR1 gene in mycorrhizal plants infected with Beet curly top Iran virus (BCTIV) was observed. Additionally, in tobacco mosaic virus infected mycorrhizal plants, several defense-related genes, such as PR proteins coding genes were decreased and it was suggested to be related to the higher infectivity of the virus [20]. This shows the relationship between viral resistance and the AM fungi effect on PR protein expression levels and may explain the change in the amount of viral infectivity in MV plants. We also found that colonization of tomato, whether after or before viral infection, can increase plant defense responses against TBSV. This may result in an increased systemic acquired resistance in the mycorrhizal plants due to the high expression of PR genes.

Another significant finding from this study was that infection of both viruses shows no clear effect on the percentage of mycorrhization in MV and M plants at the last time point. There was no significant difference in root colonization by *R. irregulare* between M and MV treatments. Similarly, Maffei et al. [11] found that tomato root mycorrhization by *Funneliformis mosseae* was not influenced by TSWV and tomato yellow leaf curl virus (TYLCV) infection. In addition, an identical level of mycorrhizal colonization in healthy mycorrhizal potato plants and infected Potato virus Y (PVY) mycorrhizal potato plants were reported by Sipahioglu et al. [44]. Among the five treatments, VM treatments showed lower colonization at 30 dpi than M and MV treatments, probably due to the delay in mycorrhizal inoculation and plant growth stage, not the effect of the virus. While this may provide a preliminary understanding of the effect of AM fungal colonization and viral infection, further investigations are needed to better understand the correlation between viral infectivity and AM colonization. Our study showed that under the same greenhouse conditions, host cultivar and mycorrhiza, the outcomes were different for ToMV and TBSV, so the control effect of AM fungi may depend on the type of virus. The interaction among virus, plant, and AM fungi is a multifaceted system when there are numerous factors such as viral pathogen type, virus lifestyle and the timing of interaction [41]. This study clearly shows this specific interaction, which results in differential regulation of PR proteins in tomato in response to TBSV and ToMV infection.

This study had some limitations. Specifically, we tested young leaves from tomato for virus accumulation and gene expression. It has been reported that the virus concentration is variable for leaves of different ages [22]. For this, any comparison between the treatments for the leaves of different ages may be incorrect. Additionally, to prevent additional stress on experimental plants used in the treatments, comparing mycorrhization only was done at the end of the experiment.

Finally, results from this study and other studies on the interaction of plant-virus and AM fungi [43,44,45] show a differential effect for AM fungi on plant virus disease development. We found that mycorrhizal symbiosis has a positive effect on tomato plant resistance to TBSV. We need to examine other tomato cultivars and AM fungal species for their interaction with TBSV infection before applying these results in the field.

## Figures and Tables

**Table 1:** Experimental design based on the time for each virus type.

Time	Control (C)	AMF (M)	AMF + Virus (MV)	Virus (V)	Virus + AMF (VM)
0 days	Seed	Seed	Seed	Seed	Seed
30 days	−	+AMF	+AMF	−	−
50 days	−	−	+Virus	+Virus	+Virus
60 days	−	−	−	−	+AMF
70 days	Leaf sampling	Leaf sampling	Leaf sampling	Leaf sampling	Leaf sampling
74 days	Leaf sampling	Leaf sampling	Leaf sampling	Leaf sampling	Leaf sampling
80 days	Leaf sampling	Leaf sampling	Leaf sampling	Leaf sampling	Leaf sampling

**Table 2:** Gene specific primers were used for real-time PCR in this study.

Gene	Primer Sequence (5′ to 3′)	Accession No.	PCR Product Size
*LePR1*	F: 5′-GCCAAGCTATAACTACGCTACCAAC-3′	DQ159948	139 bp
	R: 5′-GCAAGAAATGAACCACCATCC-3′		
Le*PR2*	F: 5′-GGACACCCTTCCGCTACTCTT-3′	M80604	81 bp
	R: 5′-TGTTCCTGCCCCTCCTTTC-3′		
Le*PR3*	F: 5′-AACTATGGGCCATGTGGAAGA-3′	Z15140	81 bp
	R: 5′-GGCTTTGGGGATTGAGGAG-3′		
*LeUBI3*	F: 5′- TCCATCTCGTGCTCCGTCT-3′	X58253	144 bp
	R: 5′-GAACCTTTCCAGTGTCATCAACC-3′		

**Table 3:** Level of fresh weight (g) and dry weight (g) in the plants infected with TBSV and ToMV.

	TBSV		ToMV	
Treatment	Fresh Weight (g) M ± SD	Dry Weight (g) M ± SD	Fresh Weight (g) M ± SD	Dry Weight (g) M ± SD
**C**	8.12 ± 0.39 ^a^	3.1 ± 0.30 ^a^	8.12 ± 0.39 ^a^	3.1 ± 0.30 ^a^
**M**	8.61 ± 0.30 ^a^	3.4 ± 0.38 ^a^	8.61 ± 0.30 ^a^	3.4 ± 0.38 ^a^
**MV**	7.18 ± 0.34 ^b^	2.07 ± 0.23 ^b^	5.48 ± 0.23 ^b^	1.11 ± 0.15 ^b^
**V**	5.93 ±0.51 ^c^	1.06 ± 0.23 ^c^	7.16 ± 0.49 ^d^	2.13 ± 0.43 ^c^
**VM**	7.08 ± 0.28 ^b^	2.01 ± 0.37 ^b^	6.13 ± 0.84 ^b^	1.16 ± 0.24 ^b^

Note. M ± SD = Mean ± Std. Deviation. Uninfected control plants without AM fungi (C), uninfected control plants with AM fungi (M) TBSV/ToMV-infected plants without AM fungi (V), TBSV/ToMV -infected plants before mycorrhiza (VM) inoculation and inoculated plants with mycorrhiza before TBSV/ToMV infection (MV). Values of each column followed by different letters indicate significant difference between the level of FW and DW treatments according to ANOVA-Bonferroni post hoc-test (*p* < 0.05). Values of each column followed by the same letters are not significantly different between the level of FW and DW treatments according to ANOVA-Bonferroni post hoc-test (*p* < 0.05). Each value represents the mean of three replicates ± SD.
